# Scalable Nanoemulsion Formation of Lipophilic Active Ingredients via Low-Energy Phase Inversion

**DOI:** 10.3390/polym18070794

**Published:** 2026-03-25

**Authors:** Ji-Hyeon Kim, Su-Hwa Son, Hye Won Lee, Jae Hun Kim, Sung-Min Kang, Chang-Hyung Choi

**Affiliations:** 1 School of Chemical Engineering, Yeungnam University, 280, Daehak-ro, Gyeongsan 38541, Gyeongbuk, Republic of Korea; zhyeon@yu.ac.kr (J.-H.K.); tnghk0314@yu.ac.kr (S.-H.S.); 2 MR Innovation Co., Ltd., 129, Alphacity 1-ro, Suseong-gu, Daegu 42251, Gyeongbuk, Republic of Korea; fidwer55@mrinno.com (H.W.L.); scottkim@mrinno.com (J.H.K.); 3 Department of Green Chemical Engineering, Sangmyung University, Cheonan 31066, Chungnam, Republic of Korea

**Keywords:** nanoemulsion, low-energy phase inversion, scalable emulsification, lipophilic active delivery, polymer-based formulation, thermal stability

## Abstract

Nanoemulsions are widely recognized as versatile delivery platforms capable of stably loading lipophilic active ingredients. Although low-energy phase inversion methods enable nanoemulsion formation under ambient and low-shear conditions, their scalability and applicability in practical formulation environments remain insufficiently validated. Here, we develop oil-in-water (O/W) nanoemulsions via a low-energy phase inversion process and systematically investigate their composition-dependent formation, scalability, and formulation stability. By precisely tuning the composition of a mixed nonionic surfactant system, monodisperse nanoemulsions with an average droplet size of ~110 nm and a polydispersity index (PDI ≤ 0.20) are reproducibly obtained under ambient, low-shear conditions. The optimized nanoemulsions maintain their nanoscale dispersion characteristics over 30 days of storage and exhibit consistent droplet size and distribution upon scale-up to 1 L. Furthermore, the nanoemulsions retain structural stability when incorporated into polymer-based formulations under various temperature conditions and repeated thermal cycling. These results demonstrate that low-energy phase inversion enables a scalable and formulation-compatible nanoemulsion platform, providing practical guidelines for industrial formulation and manufacturing of delivery systems for lipophilic active ingredients.

## 1. Introduction

Nanoemulsions are nanoscale liquid dispersions whose structural characteristics play a critical role in determining formulation stability and performance. In cosmetic- and pharmaceutical-related formulations, nanoemulsion systems are of particular interest due to their ability to accommodate diverse functional components within dispersed liquid phases [1,2,3]. However, practical implementation remains challenging, as conventional fabrication often relies on energy-intensive processes [4,5] with limited scalability beyond laboratory-scale preparation [1,6]. High-energy emulsification techniques enable precise control over droplet size [7,8] but typically require specialized equipment and substantial energy input [9,10], which restricts their direct translation to formulation development and large-scale manufacturing environments.

Low-energy emulsification methods have therefore attracted increasing attention as alternative strategies for nanoemulsion formation. Among these, phase inversion approaches exploit composition- or temperature-induced transitions in surfactant systems to generate nanoscale droplets under mild processing conditions. These processes are primarily governed by reductions in interfacial tension and rapid interfacial rearrangement near phase inversion conditions, enabling spontaneous droplet formation without the need for high shear forces [11,12,13]. The resulting nanoemulsion stability is largely dictated by interfacial physicochemical phenomena, including surfactant adsorption at the oil–water interface, steric stabilization arising from hydrated chains, and interfacial packing effects that suppress droplet coalescence [1,14,15]. Despite these advantages, most previous studies have primarily focused on demonstrating nanoemulsion formation at small laboratory scales [16]. In contrast, systematic evaluation of scalability and preservation of nanoscale dispersion characteristics under formulation-relevant conditions remains limited. In particular, it remains unclear whether low-energy phase inversion processes can maintain droplet size uniformity and stability during batch expansion, where variations in mixing conditions, residence time, and thermal history may influence interfacial dynamics and droplet structure [17,18]. For formulation-driven applications, maintaining nanoscale integrity during scale-up is critical, as even minor structural deviations can lead to phase instability and degradation of functional performance. These limitations highlight the need for experimentally grounded benchmarks that link interfacial mechanisms to scalable formulation behavior [19].

In this study, we address the critical gap between laboratory-scale nanoemulsion formation and formulation-relevant scalability. While previous studies have primarily focused on demonstrating nanoscale droplet formation, the preservation of dispersion characteristics during scale-up and under practical formulation conditions remains insufficiently understood. Here, we systematically investigate a nanoemulsion system governed by composition-driven phase inversion, with particular emphasis on scalability and formulation applicability. We optimize key formulation parameters governing droplet structure, evaluate the reproducibility of nanoscale dispersion during batch expansion, and examine structural stability within polymer-based matrices under controlled thermal conditions. By linking interfacial mechanisms to scalable formulation behavior, this work provides a practical framework for extending low-energy nanoemulsion systems beyond laboratory-scale demonstrations toward industrially relevant applications.

## 2. Materials and Methods

### 2.1. Materials

Isopropyl myristate (IPM, Sigma-Aldrich, St. Louis, MO, USA) was used as the oil phase. Bakuchiol (99%, Sunpure Extracts Private Limited, New Delhi, India) served as a model lipophilic active ingredient. The nonionic surfactants Tween 80^®^ (polyoxyethylene (20) sorbitan monooleate, Sigma-Aldrich, St. Louis, MO, USA) and Span 80^®^ (sorbitan monooleate, Sigma-Aldrich, St. Louis, MO, USA) were used as received. Glycerol (≥99%, Junsei Chemical, Tokyo, Japan) was used to prepare the aqueous phase, and all aqueous solutions were prepared using deionized (DI) water. Carbopol^®^ 940 (carbomer, polyacrylic acid, Lubrizol Corporation, Cleveland, OH, USA) was used as the base gel for ampoule formulations, and sodium hydroxide (NaOH, Sigma-Aldrich) was used for pH adjustment. Oil Red O (≥95%, Sigma-Aldrich, St. Louis, MO, USA) was used as a lipophilic dye for visual observation of the nanoemulsions.

### 2.2. Preparation of Lipophilic Active-Loaded Nanoemulsions via Low-Energy Phase Inversion

Bakuchiol-loaded nanoemulsions were prepared using a low-energy phase inversion method. The oil phase was prepared by dissolving bakuchiol in IPM at a concentration of 5 wt%. A mixed surfactant system consisting of Tween 80 and Span 80 was used, targeting an HLB value of 13. Tween 80 and Span 80 were mixed at a weight ratio of 81.3:18.7 [20]. The aqueous phase consisted of an 8 wt% glycerol solution in DI water. All components were prepared at ambient temperature without external heating. The phase inversion process was conducted under low-shear conditions. The oil phase was stirred at 300 rpm using an overhead stirrer (JS Research Inc., JSMS-070, Gongju, Republic of Korea), while the aqueous phase was continuously injected at a flow rate of 5 mL min^−1^ using a syringe pump (Harvard Apparatus, Holliston, MA, USA). The resulting nanoemulsions were used directly for droplet size analysis and formulation studies without further post-treatment and were stored at room temperature.

### 2.3. Adjustment of Surfactant HLB Values

To investigate the effect of surfactant HLB on nanoemulsion formation, mixed surfactant systems were prepared by combining Tween 80 (HLB 15.0) and Span 80 (HLB 4.3). The HLB value of each mixture was calculated using (1):

(1) $${HLB}_{mix}=f_{Tween\;80}\;\times {HLB}_{Tween\;80}\;+\;f_{Span\;80}\;\times {HLB}_{Span\;80}$$

where f represents the weight fraction of each surfactant. Nanoemulsions were prepared using identical phase inversion conditions for each HLB composition.

### 2.4. Dynamic Light Scattering (DLS) Analysis

The hydrodynamic diameter, size distribution, and polydispersity index (PDI) of the nanoemulsions were measured using dynamic light scattering (DLS), while the zeta potential was determined to evaluate the surface charge characteristics of the nanoemulsions. The hydrodynamic diameter reflects the effective size of the dispersed droplets, including the surrounding hydration layer. Samples were prepared by diluting 5 μL of nanoemulsion in 3 mL of deionized (DI) water. Each sample was thoroughly mixed prior to measurement. Measurements were performed at 25 °C using a Zetasizer Nano ZS (Malvern Instruments, Malvern, UK). Each condition was measured in triplicate (*n* = 3), and the results were reported as mean values. Zeta potential measurements were conducted using the same instrument and identical dilution conditions via electrophoretic light scattering. A disposable folded capillary cell (DTS1070) was used for zeta potential measurements instead of a standard cuvette. The electrophoretic mobility was converted to zeta potential using the Smoluchowski approximation. Stability analysis of nanoemulsions dispersed in ampoule formulations was conducted under the same dilution and measurement conditions.

### 2.5. Cryogenic Transmission Electron Microscopy (Cryo-TEM)

The microstructure and dispersion state of bakuchiol-loaded nanoemulsions were examined using cryogenic transmission electron microscopy (Cryo-TEM). Observations were performed using a Bio-TEM (Talos L120C, Thermo Fisher Scientific, Eindhoven, Netherlands) operated at an accelerating voltage of 120 kV. Samples were prepared directly from freshly prepared nanoemulsions and rapidly vitrified to preserve their native structure. Cryo-TEM images were used to evaluate droplet morphology, size distribution, and the presence of aggregation or coalescence.

### 2.6. Preparation of Nanoemulsion Ampoule Formulations and Temperature Stability Tests

Nanoemulsion-based ampoule formulations were prepared to evaluate practical formulation applicability. A 0.01 wt% Carbopol aqueous gel served as the base formulation, and the pH was adjusted to 5.5 using NaOH. The pH was measured using a pH meter (a-AB33PH, OHAUS Corporation, Parsippany, NJ, USA).

Nanoemulsions were incorporated into the Carbopol gel at 10% (*v*/*v*) and mixed at 300 rpm until a homogeneous dispersion was obtained. Temperature stability was evaluated using standard isothermal storage tests commonly employed in cosmetic formulations. Samples were stored at 4 °C, 25 °C, and 40 °C, and nanoemulsion stability was analyzed by DLS after defined storage periods. Thermal cycling tests were additionally conducted to assess resistance to temperature fluctuations. One cycle consisted of sequential exposure to 4 °C (24 h), 25 °C (24 h), 40 °C (24 h), 4 °C (24 h), and 25 °C (24 h). This cycle was repeated three times. After completion of the cycling tests, DLS measurements were performed to evaluate dispersion stability and the presence of phase separation.

## 3. Results

### 3.1. Formation of Lipophilic Active-Loaded O/W Nanoemulsions via Phase Inversion

**Figure 1:**
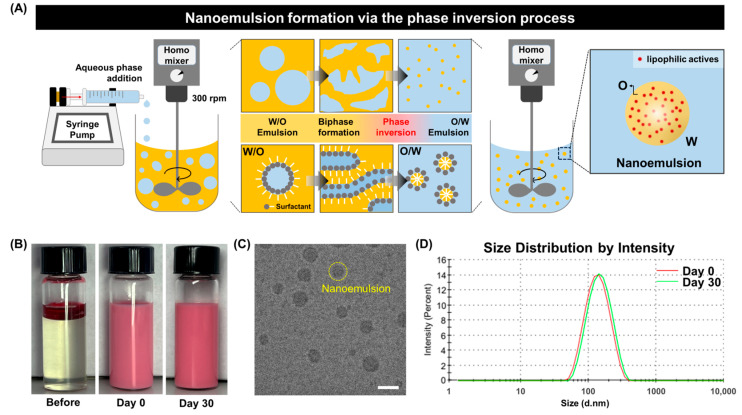
Formation and structural characteristics of nanoemulsions via low-energy phase inversion. (**A**) Schematic illustration of the phase inversion process. Arrows indicate the direction of phase inversion during emulsification. The yellow color represents the oil phase, the blue color represents the aqueous phase. (**B**) Optical images before and after emulsification. (**C**) Cryo-TEM image showing nanoscale droplets. (**D**) DLS size distributions at Day 0 and after 30 days.

To generate nanoemulsions that stably load lipophilic actives under ambient and low-shear conditions, a low-energy phase inversion process based on a mixed nonionic surfactant system is employed [21]. Isopropyl myristate (IPM) is used as the oil phase, and the hydrophilic–lipophilic balance (HLB) is precisely tuned by mixing Tween 80 and Span 80. A lipophilic active (5 wt%) is fully dissolved in the oil phase, while the aqueous phase consists of an 8 wt% glycerol solution. All components are prepared at ambient temperature without external heating. The aqueous phase is continuously injected into the stirred oil phase using a syringe pump under low-shear mixing (300 rpm). As the aqueous volume fraction gradually increases, the system undergoes a phase inversion sequence from a W/O emulsion to a biphasic state and finally to an O/W nanoemulsion (Figure 1A).

During the initial stage, a W/O emulsion forms with the aqueous phase dispersed in the oil phase [20,21]. As the aqueous volume fraction ($\varphi_{\omega }$) increases, the total interfacial area expands rapidly [11,12,18]. Under a fixed surfactant concentration, the effective surfactant coverage per unit interface decreases, accompanied by increased interfacial hydration. Consequently, the effective HLB of the surfactant system shifts from hydrophobic toward hydrophilic character [13]. Near the composition where the oil and aqueous phases become comparable in volume, the interfacial tension (γ) reaches a minimum, significantly lowering the energy barrier for droplet breakup in the biphasic region. Once the aqueous phase becomes thermodynamically favored as the continuous phase, rapid interfacial rearrangement induces phase inversion, producing uniform O/W nanoemulsions with droplet sizes in the tens to hundreds of nanometers [12,17,19,20,21]. Importantly, the droplet formation in this system is governed by composition-driven phase inversion rather than shear-induced fragmentation [1,22,23]. Unlike high-energy emulsification methods, where droplet size is strongly dependent on shear intensity, the present system relies on interfacial tension reduction and thermodynamically driven phase transition [24]. Therefore, nanoscale droplet formation can be achieved under low-shear conditions, and the role of stirring is primarily to ensure sufficient mixing rather than to determine droplet size.

In the resulting nanoemulsions, the lipophilic active remains confined within nanoscale oil droplets rather than being directly exposed to the aqueous environment (Figure 1A). This structure provides a stable platform for dispersing and protecting lipophilic actives in aqueous media [1,2,3]. To visually verify the loading of lipophilic actives within the oil droplets, a model lipophilic dye (Oil Red O) is incorporated into the oil phase (Figure 1B). Prior to emulsification, distinct phase separation is observed, whereas after phase inversion the dispersion exhibits a uniform pale pink appearance, indicating fine distribution of oil droplets within the aqueous continuous phase. The microstructure of the nanoemulsions is further characterized by cryogenic transmission electron microscopy (Cryo-TEM) (Figure 1C). The images reveal spherical nanoscale oil droplets with relatively uniform size distributions and no apparent aggregation or coalescence, confirming the structural stability of the nanoemulsions formed via the low-energy phase inversion process. This morphological observation is consistent with the DLS-derived size distributions. Droplet size characteristics are quantitatively evaluated using dynamic light scattering (DLS) (Figure 1D). Immediately after preparation, the nanoemulsions exhibit an average droplet size of approximately 110 nm with a single, monodisperse peak. The measured zeta potential (~−10 mV) indicates a near-neutral surface charge, suggesting that electrostatic stabilization is not the dominant mechanism in this system. Instead, the nanoemulsion stability is primarily governed by steric stabilization arising from the hydrated chains of nonionic surfactants, along with interfacial packing that suppresses droplet coalescence. After storage at 25 °C for 30 days, no significant change in mean droplet size or distribution profile is observed. The size variation remains within 5–10%, and the polydispersity index (PDI) stays below 0.20, satisfying the stability criteria defined in this study [1]. These results demonstrate that the nanoemulsions maintain physical stability under long-term storage conditions.

### 3.2. Evaluation of Nanoemulsion Formation Behavior and Stability as a Function of Surfactant Composition in the Phase Inversion Process

**Figure 2:**
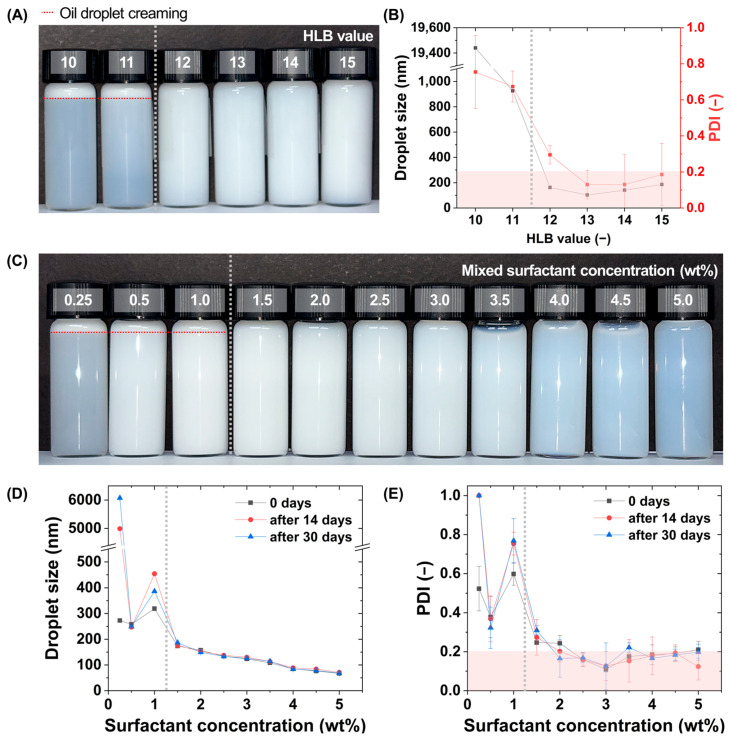
Evaluation of nanoemulsion formation and stability as a function of surfactant composition in the phase inversion process. (**A**) Photographs of nanoemulsions prepared at HLB 10–15, showing creaming at HLB 10–11 and uniform dispersion at HLB 12–15. (**B**) DLS analysis of average droplet size and PDI as a function of HLB value, showing minimum droplet size and PDI ≤ 0.20 at HLB 13. (**C**) Photographs of nanoemulsions prepared at total mixed surfactant concentrations of 0.25–5 wt%, showing creaming below 1.5 wt%. (**D**,**E**) DLS analysis of average droplet size (**D**) and PDI (**E**) at Day 0, 14, and 30 (25 °C) as a function of total mixed surfactant concentration, showing maintained nanoscale size and PDI ≤ 0.20 at ≥ 2.5 wt%. Red dashed lines indicate creaming; shaded regions denote PDI ≤ 0.20; gray dashed lines mark the transition between unstable and stable conditions.

The formation behavior and long-term dispersion stability of nanoemulsion formulations prepared by the phase inversion process were systematically evaluated as a function of surfactant composition. In particular, the HLB value and total surfactant concentration were analyzed as parameters governing phase inversion kinetics. First, to precisely control the HLB value of the surfactant system, mixed surfactants are prepared by combining the hydrophilic surfactant Tween 80 (HLB 15.0) and the lipophilic surfactant Span 80 (HLB 4.3) to cover an HLB range of 10 to 15. Each HLB value is quantitatively determined according to the calculation formula by adjusting the mass fractions of the two surfactants. Nanoemulsions are prepared under identical process conditions using each mixed surfactant composition (Figure 2A).

In general, nanoemulsions are defined as emulsion systems with droplet sizes distributed within the range of 20 to 200 nm [24,25,26]. Such nanoscale droplets exhibit kinetic stability against creaming and gravitational phase separation [1]. In addition, a polydispersity index value of 0.2 or lower is interpreted as indicative of a monodisperse distribution, indicating that the dispersed droplets exist within a relatively narrow size range [1]. DLS analysis shows distinct differences in average droplet size and PDI depending on the HLB value. Visual observation of the emulsion appearance shows that under HLB 10 and 11 conditions, a turbid upper layer forms immediately after preparation, indicating creaming. In contrast, no such layer formation is observed within the HLB 12 to 15 range. Under HLB 10 and 11 conditions, insufficient interfacial stabilization results in the formation of relatively large droplets, and as a result, oil droplets migrate upward under gravity, leading to creaming. To quantitatively verify these visual observations, DLS analysis is performed (Figure 2B). The DLS analysis results show that droplet size and PDI vary markedly with HLB value. At HLB 13, the smallest average particle size of approximately 110 nm is obtained, and the PDI is 0.2 or lower, indicating the most uniform dispersion characteristics. In contrast, at HLB 11 or lower, the average particle size exceeds approximately 800 nm and the PDI is 0.6 or higher, indicating a highly non-uniform dispersion. At HLB 14 or higher, the average droplet size increases again compared with HLB 13. It is considered that excessive hydrophilicity reduces interfacial flexibility and results in a slight increase in droplet size. These results indicate that during the phase inversion process, the hydrophilic and lipophilic balance of the surfactant regulates the curvature and interfacial tension at the oil–water interface [13]. While the HLB framework provides a practical guideline, the observed phase inversion behavior can also be qualitatively interpreted within the HLD framework, where the optimal condition is expected to correspond to a near-balanced interfacial state (HLD ≈ 0). Under HLB 13 conditions, the O/W interface most efficiently enables droplet disruption and rapid interfacial rearrangement.

Accordingly, HLB 13 is selected as the optimal surfactant condition and is fixed for all subsequent experiments. Next, to evaluate the effect of total surfactant concentration on nanoemulsion stability, nanoemulsions are prepared within the range of 0.25 to 5 wt% (Figure 2C). Visual observation shows that at total surfactant concentrations of 1 wt% or lower, upper layer formation occurs immediately after preparation and a uniform nanoemulsion is not formed. Notably, a non-monotonic trend is observed in the intermediate concentration range (0.5–1 wt%), where both droplet size and PDI increase compared to lower concentrations. This behavior can be interpreted as a transitional regime during nanoemulsion formation. In this regime, the rate of interfacial area generation during phase inversion exceeds the rate of surfactant adsorption, resulting in incomplete interfacial coverage. Consequently, transient droplet coalescence occurs, leading to larger droplet sizes and broader size distributions. In contrast, at concentrations of 1.5 wt% or higher, nanoemulsions with uniform appearance are formed without layer separation, confirming that this concentration range is suitable for nanoemulsion formation via the phase inversion-based low-temperature process.

In addition, as the surfactant concentration increases, the nanoemulsions gradually exhibit a bluish appearance. This phenomenon is attributed to a decrease in average particle size to approximately 100 nm or lower, whereby the size of the dispersed oil droplets becomes comparable to the wavelength of visible light and Rayleigh scattering becomes dominant over Mie scattering. As a result, shorter wavelengths are scattered more strongly and the nanoemulsion appears blue [4,20,23,27,28]. The prepared formulations are stored at room temperature, and particle size and PDI are measured immediately after preparation at day 0 and after 14 and 30 days to evaluate initial and long-term stability (Figure 2D,E). Long-term stability analysis shows that at total surfactant concentrations of 1 wt% or lower, the variation in average particle size increases to approximately 20 percent or higher during storage, and the PDI also increases to 0.2 or higher, indicating that dispersion stability is not maintained. At 1.5 to 2 wt%, the variation in average particle size remains within 8 percent, but the PDI increases to 0.2 to 0.4, indicating a decrease in particle uniformity. In contrast, at 2.5 wt% or higher, the variation in average particle size remains within approximately 8% over 30 days of storage and the PDI is maintained at 0.20 or lower, demonstrating long-term stable dispersion.

As the surfactant concentration increases, the average particle size gradually decreases. This is attributed to the increased surfactant concentration enhancing interfacial coverage at the oil–water interface and improving the rate of interfacial rearrangement during phase inversion, thereby effectively suppressing transient droplet aggregation [12,13,17,23,29]. Among the tested conditions, 3 wt% is determined to be the practical optimal concentration. At this concentration, minimal droplet size variation and stable monodisperse characteristics are simultaneously achieved, whereas no further improvement in structural stability is observed at 4 wt% or higher. This indicates that excess surfactant remains in the continuous phase without contributing to additional droplet stabilization. Therefore, the composition identified under the present experimental conditions was selected as the reference formulation for subsequent scale-up studies and cosmetic application evaluation.

### 3.3. Evaluation of Dispersion Characteristics and Stability upon Scale-Up of Phase Inversion-Based Nanoemulsions

Previous studies on nanoemulsions prepared via phase inversion processes have largely been limited to small-scale laboratory conditions, and systematic verification of whether identical dispersion structures and reproducibility are maintained upon increasing the production volume remains limited [1,21]. The scale-up to 1 L is considered as a pilot-scale demonstration bridging laboratory-scale experiments and industrial production. To minimize hydrodynamic variability, the mixing conditions and injection rate were maintained consistent across all batch volumes. More importantly, droplet formation in this system is governed by composition-driven phase inversion rather than shear-dependent fragmentation. Therefore, the droplet size and distribution are primarily dictated by interfacial physicochemical conditions, and are not strongly affected by hydrodynamic variations once sufficient mixing is achieved.

For low-energy processes, potential variations in dispersion characteristics due to changes in process conditions or expansion of mixing scale have consistently been raised as concerns [18,19]. Accordingly, in this study, the optimal compositional conditions derived in Figure 2 (HLB 13, total surfactant concentration 3 wt%) are maintained, and the effect of increasing production volume on the dispersion characteristics and storage stability of the nanoemulsions is systematically evaluated.

**Figure 3:**
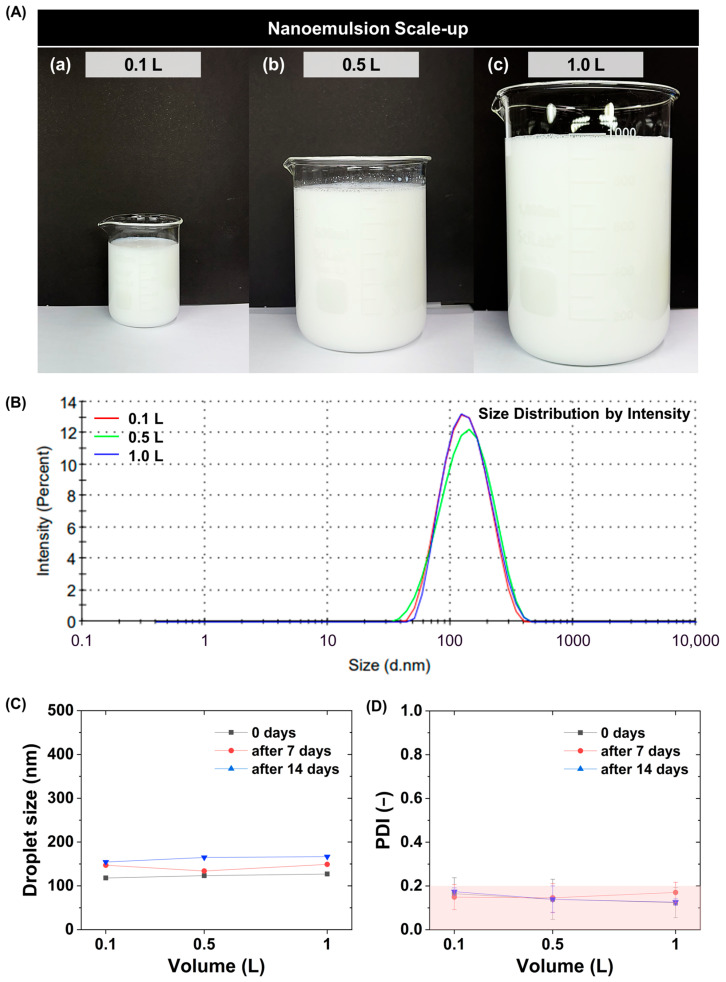
Evaluation of nanoemulsion dispersion characteristics during batch-scale expansion. (**A**) Photographs of nanoemulsions prepared at batch volumes of (**a**) 0.1, (**b**) 0.5, and (**c**) 1.0 L under identical conditions (HLB 13 and total mixed surfactant concentration of 3 wt%). (**B**) DLS droplet size distributions showing comparable nanoscale droplets (~110 nm) across all volumes. (**C**,**D**) DLS analysis of average droplet size (**C**) and PDI (**D**) at Day 0, 7, and 14 (25 °C) as a function of batch volume, showing stable droplet size and PDI ≤ 0.20 under scale-up conditions. The red shaded region denotes conditions with PDI ≤ 0.20.

Nanoemulsions are prepared by stepwise scaling up the batch volume to 0.1, 0.5, and 1.0 L while maintaining identical composition and stirring conditions (Figure 3A). Under all conditions, a visually uniform appearance is preserved, and no phase separation or aggregation is observed. To quantitatively compare the dispersion characteristics as a function of production volume, DLS analysis is conducted (Figure 3B). The results show that under 0.1, 0.5, and 1.0 L conditions, the average particle size is consistently formed at approximately 110 nm, and the particle size distribution curves exhibit nearly overlapping profiles. This clearly demonstrates that within the evaluated volume range, increasing the production volume does not induce any significant changes in average particle size or distribution characteristics. This indicates that the nanoscale dispersion structure is preserved even when the batch size is expanded to the liter scale. In particular, the absence of significant changes in droplet size suggests that the droplet formation mechanism in this system is governed not primarily by shear-dependent fragmentation, but rather by composition-driven phase inversion. To evaluate the storage stability of the scaled-up nanoemulsions, particle size and PDI are analyzed for each batch condition (0.1, 0.5, and 1.0 L) immediately after preparation (0 day) and after storage at room temperature for 7 and 14 days (Figure 3C,D).

Across all batch conditions, the variation in average droplet size remains within approximately 10%, and the PDI is consistently maintained at ≤0.20. Notably, even when the production volume increases tenfold from 0.1 L to 1.0 L, no systematic increase in particle size variation is observed. PDI analysis likewise confirms that values remain ≤0.20 throughout the storage period for all volume conditions. This indicates that the scaled-up nanoemulsions stably maintain a uniform particle size distribution without aggregation or dispersion collapse. Comparative analysis across volumes further shows that the average particle size (approximately 110 nm) and PDI (≤0.20) remain nearly identical within the 0.1–1.0 L range. Collectively, these results demonstrate that phase inversion-based nanoemulsions can be reproducibly manufactured at the liter scale, experimentally confirming that the composition-driven phase inversion mechanism maintains high compositional robustness during batch scale-up.

### 3.4. Applicability of Phase Inversion Nanoemulsions in Polymer-Based Ampoule Formulations and Temperature-Dependent Structural Stability

**Figure 4:**
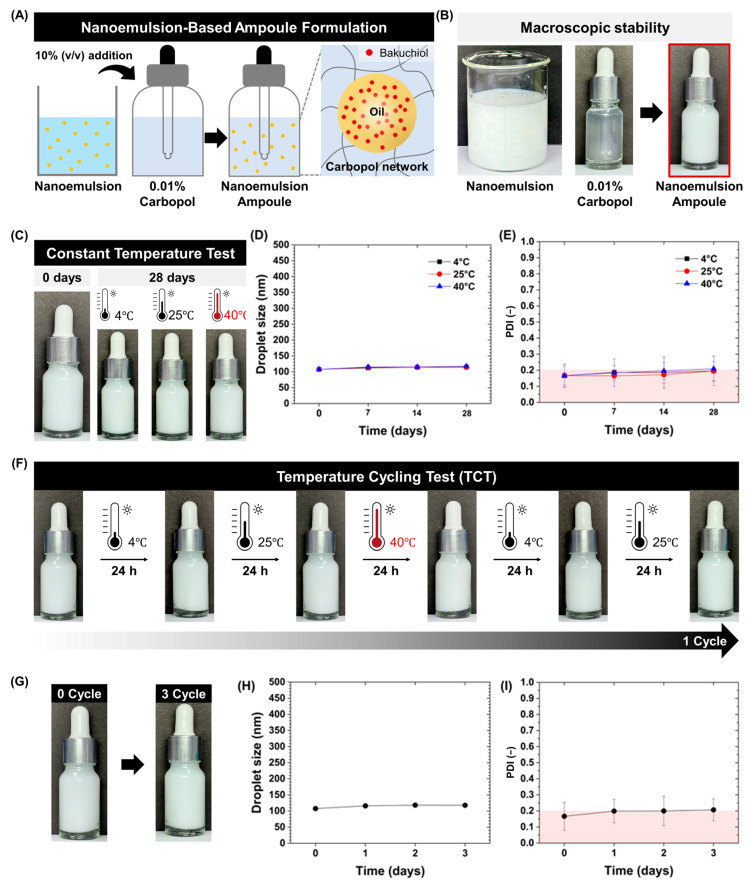
Formulation applicability and temperature stability of phase-inverted nanoemulsions within a polymer-based matrix. (**A**) Schematic illustration of the preparation of a nanoemulsion ampoule by incorporating lipophilic active-loaded nanoemulsions into a 0.01 wt% Carbopol matrix at 10% (*v*/*v*). Arrows indicate the addition of nanoemulsions into the Carbopol matrix and the subsequent formation of the ampoule formulation. (**B**) Photographs of the pristine nanoemulsion, 0.01 wt% Carbopol base, and nanoemulsion-loaded ampoule. (**C**–**E**) DLS analysis of average droplet size (**D**) and PDI (**E**) during constant temperature storage at 4, 25, and 40 °C for 28 days, showing maintained droplet size (~110 nm) and PDI ≤ 0.20. (**F**) Temperature cycling test (4 → 25 → 40 °C, 24 h each per step). (**G**) Photographs of the ampoule before and after temperature cycling. (**H**,**I**) DLS analysis of average droplet size (**H**) and PDI (**I**) following temperature cycling, showing preserved nanoscale droplet size and PDI ≤ 0.20. The red shaded region denotes conditions with PDI ≤ 0.20.

Beyond scalable production, practical utility ultimately depends on compatibility with real formulation matrices. The performance of phase inversion nanoemulsions is therefore systematically examined within polymer-based ampoule formulations under varying temperature conditions. Nanoemulsion ampoules are prepared by incorporating the nanoemulsions into a 0.01 wt% Carbopol gel (pH 5.5) at a ratio of 10% (*v*/*v*) (Figure 4A). Mixing is performed at 300 rpm to obtain homogeneous dispersions. As shown in Figure 4B, the resulting ampoules exhibit a uniform milky appearance and remain macroscopically homogeneous without localized aggregation or phase separation after incorporation into the polymer matrix. This observation indicates that the nanoscale oil droplets containing lipophilic actives remain stably dispersed within the polymer network.

To simulate practical storage conditions, the nanoemulsion ampoules are stored at 4 °C, 25 °C, and 40 °C for up to 28 days, and isothermal stability is evaluated (Figure 4C). No macroscopic changes, such as creaming, sedimentation, or phase separation, are observed under any temperature condition, indicating robust physical stability of the formulations across a wide temperature range. Quantitative structural stability is assessed using DLS (Figure 4D,E). The initial average droplet size is approximately 110 nm, and after 28 days of storage the overall size variation remains within 10% under all temperature conditions. These minor changes are attributed to limited interfacial rearrangement and weak droplet interactions within the polymer matrix, while irreversible coalescence is not detected. The polydispersity index remains below 0.20 throughout the storage period, even at elevated temperature, confirming the preservation of monodisperse droplet structures. These results suggest that the mixed nonionic surfactant system provides effective steric stabilization in polymeric environments.

The resistance of the formulations to rapid temperature fluctuations is further evaluated using thermal cycling tests (TCT) (Figure 4F). One cycle consists of sequential exposure to 4 °C, 25 °C, 40 °C, 4 °C, and 25 °C for 24 h at each step, and the cycle is repeated three times. As shown in Figure 4G, the ampoules retain uniform macroscopic appearances without visible deterioration after repeated cycling. DLS measurements (Figure 4H,I) reveal only slight increases in droplet size with increasing cycle number, while the overall size variation remains within 5–10%. The PDI also remains below 0.20 under all cycling conditions, indicating that monodisperse structures are maintained despite repeated thermal stress. Collectively, the lipophilic active-loaded nanoemulsions produced via phase inversion exhibit excellent structural durability within polymer-based formulations under prolonged thermal stress and repeated temperature fluctuations. These findings demonstrate the practical potential of this nanoemulsion platform for real cosmetic formulation environments.

## 4. Conclusions

This study establishes a scalable oil-in-water nanoemulsion platform capable of loading lipophilic active ingredients via a low-energy phase inversion process. Precise interfacial control using a mixed nonionic surfactant system enables the reproducible formation of monodisperse nanoemulsions under ambient, low-shear conditions, while maintaining long-term structural stability even at minimal surfactant concentrations. This approach effectively overcomes key limitations of conventional high-energy nanoemulsification methods, including process complexity and potential degradation of sensitive components. The optimized formulation maintains consistent dispersion characteristics and structural stability during stepwise scale-up, demonstrating that the phase inversion process can be extended beyond laboratory-scale preparation toward industrially relevant production volumes. Furthermore, beyond scalable production, this study experimentally demonstrates formulation-level applicability by incorporating the nanoemulsions into polymer-based systems and systematically evaluating their structural stability under various temperature conditions and thermal cycling. Collectively, these findings demonstrate that low-energy phase inversion provides a practical and scalable nanoemulsion platform that integrates process expandability with formulation compatibility. This work provides a practical framework for designing and manufacturing delivery systems for lipophilic active ingredients across a broad range of industrial applications, including cosmetic formulations.

## Data Availability

The original contributions presented in this study are included in the article/Supplementary Material. Further inquiries can be directed to the corresponding authors.

## Supplementary Materials

The following supporting information can be downloaded at: https://www.mdpi.com/article/10.3390/polym18070794/s1, Figure S1: Zeta potential distribution of nanoemulsions prepared under optimized conditions (HLB 13, 3 wt% mixed surfactant concentration).
