# Supplementary material for: Scalable Nanoemulsion Formation of Lipophilic Active Ingredients via Low-Energy Phase Inversion
Source: Polymers (Basel). 2026 Mar 25;18(7):794. doi: 10.3390/polym18070794 (PMC13074364; doi:10.3390/polym18070794)
Supplement: Supplementary file 1 [file polymers-18-00794-s001.zip › polymers-4201067-supplementary.pdf]

Supplementary material for

Scalable Nanoemulsion Formation of Lipophilic Active Ingredients via Low-Energy Phase Inversion

Ji-Hyeon Kim [1], Su-Hwa Son [1], Hye Won Lee [2], Jae Hun Kim [2], Sung-Min Kang [3,*] and Chang-Hyung Choi [1,*]

[1] School of Chemical Engineering, Yeungnam University, 280, Daehak-ro, Gyeongsan 38541, Gyeongbuk, Republic of Korea; zhyeon@yu.ac.kr (J.-H.K.); tnghk0314@yu.ac.kr (S.-H.S.)
[2] MR Innovation Co., Ltd., 129, Alphacity 1-ro, Suseong-gu, Daegu 42251, Gyeongbuk, Republic of Korea; fidwer55@mrinno.com (H.W.L.); scottkim@mrinno.com (J.H.K.)
[3] Department of Green Chemical Engineering, Sangmyung University, Cheonan 31066, Chungnam, Republic of Korea
* Correspondence: smkang@smu.ac.kr (S.-M.K.); chchoi@yu.ac.kr (C.-H.C.)

S1. Assessment of surface charge characteristics of nanoemulsions by zeta potential measurement.

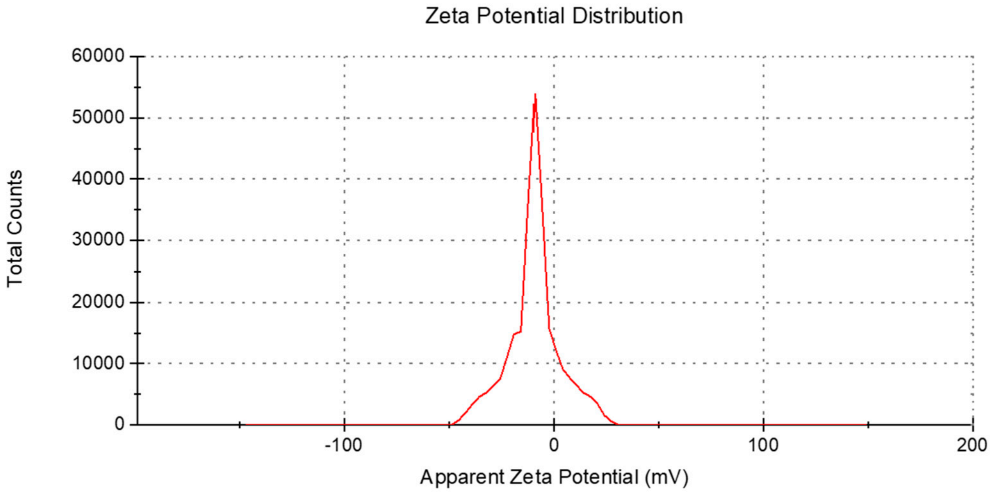

Figure S1. Zeta potential distribution of nanoemulsions prepared under optimized conditions (HLB 13, 3 wt% mixed surfactant concentration).
